# Suitability of Ultra-Short-Term Heart Rate Variability in Military Trainees

**DOI:** 10.3390/healthcare8040409

**Published:** 2020-10-17

**Authors:** Mubarak J. Alalyan, Shaea A. Alkahtani, Syed Shahid Habib, Andrew A. Flatt

**Affiliations:** 1 King Fahd Security College, Riyadh 11461, Saudi Arabia; m4.olyan@gmail.com; 2 Department of Exercise Physiology, College of Sport Sciences and Physical Activity, King Saud University, Riyadh 11451, Saudi Arabia; shalkahtani@ksu.edu.sa; 3 Department of Physiology, College of Medicine, King Saud University, Riyadh 12372, Saudi Arabia; sshahid@ksu.edu.sa; 4 Department of Health Sciences and Kinesiology, Biodynamics and Human Performance Center, Georgia Southern University—Armstrong, Savannah, GA 31419, USA

**Keywords:** autonomic, parasympathetic, body composition, fitness, cardiovascular

## Abstract

We aimed to (a) evaluate the agreement between ultra-short-term and criterion resting heart rate variability (HRV) measures in military trainees, and (b) compare associations between HRV recording lengths and body composition. HRV recordings were performed for 10 min in 27 military male students. Mean RR interval, the root-mean square of successive differences (RMSSD), RMSSD:RR interval ratio, standard deviation of normal-to-normal RR intervals (SDNN), and SDNN:RR interval ratio were determined from the last 5 min of the 10-min recording and considered the criterion. Parameters were also recorded in successive 1-min epochs from the 5-min stabilization period. No differences were observed between criterion values and any of the 1-min epochs (*p* > 0.05). Effect sizes ranged from −0.36–0.35. Intra-class correlations ranged from 0.83–0.99. Limits of agreement ranged from 38.3–78.4 ms for RR interval, 18.8–30.0 ms for RMSSD, 1.9–3.1 for RMSSD:RR, 24.1–31.4 ms for SDNN, and 2.5–3.0 for SDNN:RR. Body fat% was associated (*p* < 0.05) with all HRV parameters at varying time segments. A 1-min HRV recording preceded by a 1-min stabilization period seems to be a suitable alternative to criterion measures. Ultra-short procedures may facilitate routine HRV tracking in tactical populations for status-monitoring purposes.

## 1. Introduction

Imbalanced autonomic nervous system functioning is observed in a variety of cardiovascular [1], metabolic [2], and neurocognitive [3] disorders. This has generated greater interest in identifying convenient, non-invasive markers of ANS status. Heart rate variability (HRV) is regarded as such a marker, reflecting autonomic regulation of cardiac cycle intervals. Traditional acquisition procedures for establishing short-term HRV in clinical or laboratory settings involve a 5-min RR interval recording preceded by a 5-min stabilization period. Research from the field of sports medicine [4], and more recently, cardiovascular medicine [5], have revealed that periodic HRV assessment is likely insufficient for practical use. Rather, near-daily recordings are preferred to create an ongoing HRV profile, enabling quantification of weekly averaged values and the magnitude of dispersion across days.

To accommodate a high frequency of HRV assessment, shorter HRV acquisition procedures have recently been investigated. Time domain HRV parameters derived from ultra-short-term RR interval recording epochs of only 1 min have been proposed as valid surrogates for 5-min criterion epochs among endurance athletes [6,7], team-sport athletes [8,9,10,11], healthy adults [7,12,13], and diabetics [14]. However, some of these investigations have received criticism for lacking statistical rigor [15]. Others have methodological inconsistencies related to whether a pre-recording stabilization period preceded RR interval acquisition, and whether selection procedures for the 1-min epoch were random or systematic. Perhaps the greatest limitation of previous work is that associations between HRV parameters (ultra-short and criterion measures) and other markers of health status have not been assessed. HRV is often associated with body composition [16], a key health marker that is modifiable with lifestyle intervention for improving ANS regulation [17,18]. Thus, validation of ultra-short-term HRV measures require further research to determine if they provide similar associations as criterion measures with health status markers such as body composition.

The suitability of ultra-short-term HRV has yet to be investigated in military populations. Whether previous support for ultra-short-term HRV in healthy and athletic populations is generalizable to tactical personnel is unclear. Though similar in age and physical characteristics to healthy or athletic adults, military personnel are exposed to unique events and activities that provoke substantial physiological and psychological strain. For example, intense physical training, frequent exposure to mental stress, and periodic conditions of sleep, water, and caloric restriction create unique circumstances that alter resting-state HRV among soldiers [19,20,21]. Moreover, HRV has been used to predict the occurrence of post-traumatic stress disorder following combat deployment [22], as well as to monitor the effects of therapeutic interventions on ANS activity [23]. Thus, HRV tracking has a variety of applications among this population, warranting further investigation into expedient recording procedures that facilitate frequent assessment. Therefore, the aim of current study was to investigate the agreement between ultra-short-term and criterion HRV measures in military trainees. A secondary aim was to compare associations between HRV recording lengths and body composition. We hypothesized that HRV parameters derived from a 1-min epoch preceded by only 1 min of stabilization would be comparable to criterion 5-min epochs. Additionally, ultra-short-term and criterion HRV parameters would be similarly associated with body composition markers.

## 2. Materials and Methods

### 2.1. Study Design

This was a cross-sectional study that examined the agreement between ultra-short-term and criterion HRV. Associations between HRV parameters and markers of body composition were also quantified.

### 2.2. Participants

Participants were military student men at the King Fahd Security College in Riyadh, Saudi Arabia (n = 27, age = 21.1 ± 1.7 years, height = 175.2 ± 4.9 cm, weight = 68.7 ± 6.7 kg, body mass index (BMI) = 22.1 ± 2.1 kg/m^2^, systolic (SBP) and diastolic (DBP) blood pressure = 122.1 ± 4.5 mmHg and 81.2 ± 2.7 mmHg, respectively). Recruitment flyers were posted on bulletin boards of the students’ guild in the college. Volunteers who expressed interest to participate in the study were met and the study procedure was explained. Inclusion criteria were to be non-smokers and be free from any current musculoskeletal injuries. The list of volunteers was approved by the Director/Major of students to allow them to participate in the study. The study protocol was reviewed by the Research and Education Center in the college and was approved by the institutional review board (IRB) of King Saud University (IRB No. E-19-4240) following the guidelines set forth in the Declaration of Helsinki. All participants understood their rights and responsibilities and signed a consent form of voluntary participation.

Participants kept their military lifestyle in the college during the period of data collection. They followed a strict and regimented schedule for sleep, wake, and meal times. Their theoretical and practical training included the study and practice of military skills (e.g., weapons training, tactical simulations, and mental skills), physical fitness (e.g., daily 30-min jogging and sport activities), and academic education (e.g., psychological studies and military laws). Participants were allowed to leave the college on weekends only. Volunteers were asked to refrain from excess caffeine consumption and vigorous or prolonged exercise training on the day prior to laboratory measures, and to come to the laboratory in the morning after an overnight fast. Laboratory measures were undertaken in the Exercise Physiology Laboratories at the College of Sport Sciences and Physical Activity, KSU, Riyadh.

### 2.3. Laboratory Measures

Upon arrival, participants were seated for 5 min preceding two measures of SBP and DBP using an automatic brachial sphygmomanometer (Omron HEM-7121, Omron Healthcare manufacturing, Kyoto, Japan). Values are provided in the “Participants” section and are included for descriptive purposes. Body composition and HRV assessments were subsequently performed.

#### 2.3.1. Body Composition

Anthropometric variables including height and weight were recorded. Body mass index (BMI) was calculated by dividing participants’ weight in kilograms by their height in meters squared. A multi-frequency bioelectrical impedance analysis (BIA) device (MC-980MA, Tanita Corporation, Tokyo, Japan) was used to measure body composition [24,25]. Participants were asked to stand barefoot on the scale and hold the handles while slightly abducting their arms. After approximately 30 s, the device printed the output including the analysis of fat mass and fat-free mass in absolute (kg) and relative (%) units.

#### 2.3.2. Heart Rate Variability

The methodology for electrocardiograph preparation and recording followed our standard operating procedures in the Laboratory of Exercise Physiology, which has been previously described [26]. Following body composition assessment, participants assumed a supine position on an examination table. A computerized ECG data acquisition device (1000 Hz, PL3516 PowerLab 16/35, ADInstruments Pty Ltd., New South Wales, Australia) with 16 analog input channels was used to perform a 10-min RR interval recording while a researcher monitored signal quality. Customized software (LabChart v. 8.1.13 Windows, ADInstruments Pty Ltd. New South Wales, Australia) was used to process ECG data and compute time-domain variables. Cardiac cycles were classified as being normal, artifact, or ectopic beat based on automatic analysis of waveform morphology performed by the software. Artifacts and ectopic beats were excluded from calculations (≤1.6% RR interval removal per sample). HRV parameters were derived from each 1-min segment of the traditional 5-min stabilization period (i.e., min 0–1, min 1–2, min 2–3, min 3–4, min 4–5) for comparison to the 5-min criterion segment (i.e., min 5–10). Parameters recorded for analysis were the mean RR interval, the standard deviation of the mean RR interval (SDNN), and the root-mean-squared difference of successive RR intervals (RMSSD). RMSSD and SDNN were subsequently divided by the mean RR interval and multiplied by 100 to compute the RMSSD: RR ratio and SDNN: RR ratio. Ratio values have been suggested to correct for the confounding effects of basal HR on HRV indices [27].

### 2.4. Statistical Analysis

Values are reported as mean ± standard deviation (SD). Shapiro-Wilks tests confirmed the assumption of normality for all outcome variables (*p* > 0.05). One-way analysis of variance for repeated measures were used to compare time segments for each HRV parameter. If Mauchley’s test indicated that the assumption of sphericity was violated, Greenhouse–Geisser-corrected values were obtained. Post hoc analyses were conducted with Bonferonni pairwise comparisons. Cohen’s d effect sizes (ES) were used to determine standardized difference between time-points. ES values were qualitatively interpreted as trivial (<0.20), small (<0.60), moderate (<1.2), large (<2.0), and very large (≥2.0) [28]. Intra-class correlations (ICC) were performed to assess the level of absolute agreement across time-points for each HRV parameter. ICC’s were qualitatively interpreted as small (<0.3), moderate (<0.5), large (<0.7), very large (<0.9), and nearly perfect (>0.9) [28]. The typical error and the mean bias ± 95% limits of agreement (1.96 SD) were calculated for each 1-min segment relative to the criterion. Pearson’s correlations were used to quantify associations between HRV parameters and markers of body composition. *p* values < 0.05 were considered statistically significant. Analyses were performed with JASP (Version 0.10.2, University of Amsterdam, Amsterdam, The Netherlands) and SPSS 25 (IBM Corporation, New York, NY, USA).

## 3. Results

Significant main effects of time-segment were observed for RMSSD (*p* = 0.007) and RMSSD: RR (*p* = 0.022). Post-hoc analyses showed that RMSSD and RMSSD: RR from min 0–1 were different from min 3–4 (*p* < 0.05). No differences were observed between criterion values and any of the 1-min segments for all HRV parameters (all *p* > 0.05). ES ranged from trivial–small for all comparisons. ICC’s ranged from very large–nearly perfect (ICC’s ≥ 0.83). Limits of agreement ranged from 38.3–78.4 ms for RR, 18.8–30 ms for RMSSD, 1.9–3.1 for RMSSD: RR, 24.1–31.4 ms for SDNN, and 2.5–3.0 for SDNN: RR. Values and comparison statistics are presented in Table 1.

**Figure 1:**
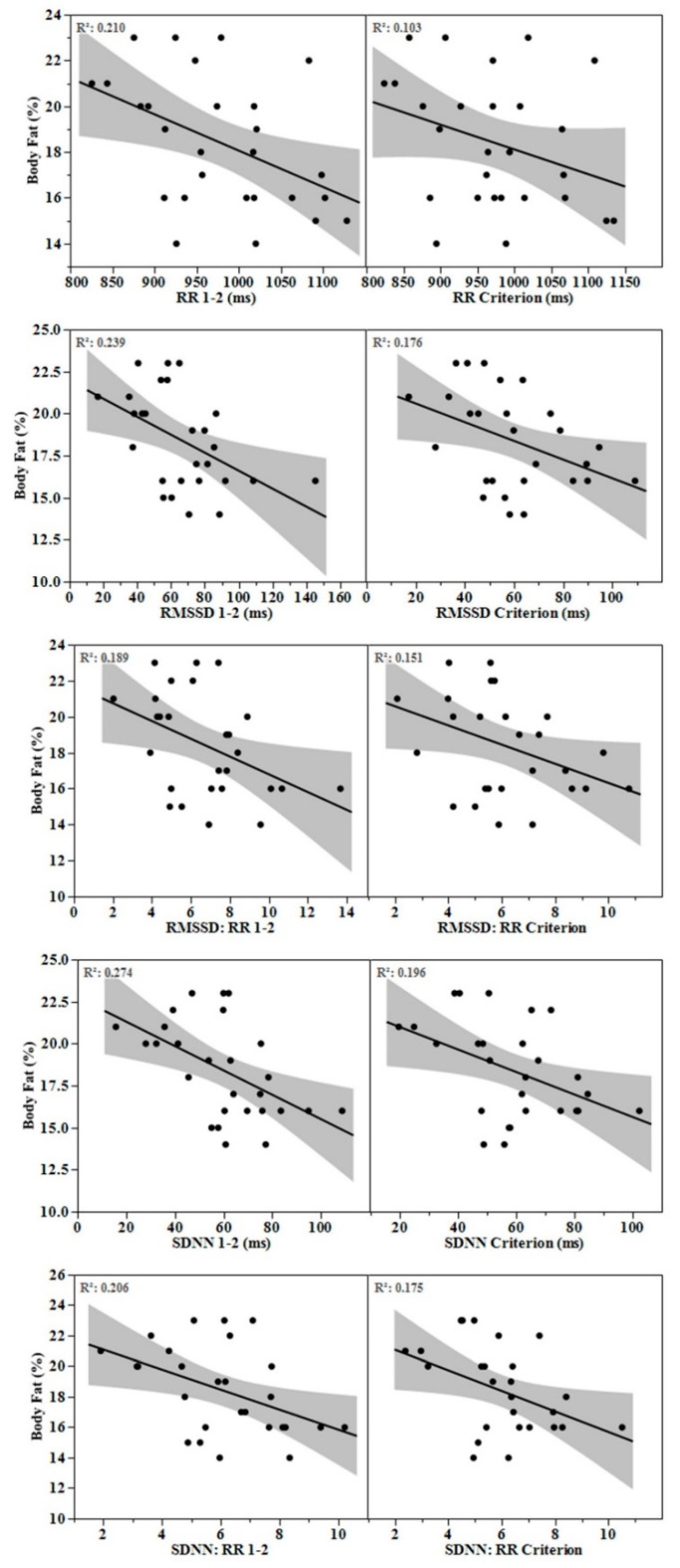
Scatterplots featuring association between body fat percentage and heart rate variability parameters derived from the 1–2 min and criterion segments.

Mean ± SD for body fat percentage, fat mass, and fat-free mass were 18.4 ± 2.9%, 12.8 ± 2.5 kg, and 56.5 ± 4.7 kg, respectively. Body fat percentage and fat mass were associated (*p* < 0.05) with all HRV parameters at varying time segments, most consistently with min 1–2 (Table 2). Scatterplots comparing associations between body fat percentage and HRV parameters derived from min 1–2 and the criterion segments are displayed in Figure 1. No associations were observed between fat-free mass and HRV parameters.

## 4. Discussion

The purpose of this study was to investigate the agreement between ultra-short-term and criterion HRV measures in military trainees. A secondary aim was to determine if ultra-short-term epochs were suitable alternatives to the criterion for drawing associations with body composition. We found no significant differences, trivial to small ES, and very large—nearly perfect ICC’s when comparing successive 1-min segments derived from the stabilization period to the criterion. HRV parameters from a majority of recording epochs were significantly associated with body fat markers.

In agreement with the current findings, investigations in athletes and healthy young adults indicate that a 5-min stabilization phase prior to HRV recordings may be excessive. Botek et al. [29] found that spectral HRV parameters during 5 min of supine rest and 5 min of standing were not different (*p* > 0.05) and strongly correlated (*p* < 0.05) when comparing values obtained following 5 or 1 min of stabilization in healthy young adults (n = 28). In collegiate endurance athletes (n = 20), no differences (all *p* = 0.99), trivial ES’s, small TE’s (TE = 0.10–0.16), near perfect ICC’s (ICC = 0.92–0.97), and narrow limits of agreement were found when comparing sequential 1 min segments from a 5-min stabilization period to the criterion for supine natural logarithm (Ln) RMSSD [6]. Very similar results have been reported for LnRMSSD [10,30] and LnRMSSD: RR [8] in team-sport and endurance athletes derived from seated recordings. In disagreement with our findings, one study found that 90 s of stabilization time was needed before a 1-min supine RMSSD measure was acceptably similar to the criterion among 30 healthy young men [7]. To our knowledge, the current investigation is the first to report the time-course for stabilization of SDNN.

Following stabilization periods of at least 5 min, numerous studies have reported that ultra-short-term epochs are suitable alternatives to criterion measures in a variety of populations. After 10 min of stabilization, Munoz et al. [31] compared RMSSD and SDNN from three consecutive 10-s samples, a 30-s sample and a 120-s sample derived from a 5-min finger pulse interval recording to the criterion (n = 3387 middle aged adults). Agreement progressively improved from the first 10 s sample to the 120 s sample. Similarly, in athletes (n = 23), it was found that following a 5-min stabilization period, randomly selected 10-, 30-, and 60-s epochs of resting supine LnRMSSD demonstrated acceptable agreement with the criterion (no significant differences, trivial–small ES, very large–near perfect ICC’s), particularly for the 60-s epoch [9]. Acceptable levels of agreement between randomly selected 1-min LnSDNN and the criterion have also been reported following a 5-min stabilization period [32]. Nussinovitch et al. [12] reported stronger ICC’s for 60 vs. 10 s randomly selected RMSSD and SDNN samples from the criterion. Our results add a practical component to the existing body of research by showing that resting ultra-short-term RMSSD and SDNN recordings of 60 s seem to be valid surrogates to criterion measures using a minimal stabilization period among military trainees.

Significant associations between ultra-short-term HRV parameters and body fat percentage were expected based on previous findings obtained with longer HRV recording epochs. For example, BIA-derived body fat mass was inversely associated with 24-h mean RR interval, RMSSD, and SDNN (all *p* < 0.05) among 68 adult women [33]. Similarly, BIA-derived body fat percentage was inversely associated with traditional short-term (5-min recording following a 5-min stabilization) RMSSD and SDNN (*p* < 0.05) among 41 health adults [34]. In addition, daytime RMSSD and SDNN were each significantly associated (*p* < 0.05) with dual-energy x-ray absorptiometry-derived (DEXA) body fat percentage in middle-aged men with metabolic syndrome (n = 97) [35]. A novel finding from the current study was that ultra-short-term measures were associated with body fat characteristics among a homogenous sample of military trainees. Moreover, Criterion RMSSD and SDNN remained associated with body fat characteristics after correction for the corresponding RR interval, as did varying ultra-short-term segments. This finding would indicate that the associations between body fat and HRV cannot fully be explained by the prevailing heart rate.

Associations between HRV and fat-free mass reported in the previous literature are less consistent. For example, in agreement with our findings, a study involving 360 police officers reported low r values between vagal-mediated HRV (supine criterion recordings) and DEXA-derived markers of fat-free mass (r = −0.148–−0.035) [36]. Contrasting with our findings, DEXA-derived fat-free mass was positively associated (*p* < 0.05) with supine RMSSD (r = 0.456–0.526) and SDNN (r = 0.571–0.617) in 28 competitive swimmers and 21 non-athletic healthy adults [37]. Thus, how lean tissue quantity influences cardiac-autonomic regulation in a variety of populations requires further investigation. Nevertheless, the current findings indicate that criterion and ultra-short-term values revealed similar null associations between resting HRV parameters and fat-free mass.

When determining how many minutes of stabilization to adopt prior to an ultra-short-term HRV recording, practitioners should consider the magnitude of difference relative to the criterion as well as potential differences in associations with clinical health markers. Krejčí et al. reported progressive reductions in the mean bias relative to the criterion with each additional minute of stabilization for RMSSD [7]. Likewise, we observed small ES’s (0.21–0.35) between RMSSD parameters and the criterion from the first and second minute of recording (i.e., min 0–1 and 1–2, respectively, Table 1). For SDNN parameters, small ES (0.34–0.36) were observed only between the 2–3-min segment and the criterion. This indicates that although non-significantly different from the criterion, a small amount of minute-to-minute variation in HRV may occur. However, associations with body fat characteristics were not improved with longer stabilization periods (Table 2, Figure 1). In fact, HRV parameters obtained following only 1 min of stabilization provided the most consistent associations with body fat markers. Though this finding may have been fortuitous, it suggests that the level of minute-to-minute variation in RMSSD and SDNN may not be clinically relevant. Future research is needed to support this finding with other health status markers.

This study was limited by the cross-sectional study design, sample size, use of only supine recordings, and lack of additional markers of health status. Future investigations should include repeated HRV measures in various positions (i.e., seated, standing), in various conditions (e.g., pre-, during, and post-deployment), and along with indicators of mental, metabolic, and cardiovascular health. Thus, whether ultra-short-term HRV measures are sensitive to adaptive or maladaptive responses to military operations or therapeutic interventions is an important next step for future research.

## 5. Conclusions

The current findings provide preliminary support for the use of resting ultra-short-term HRV parameters in military personnel. One-min samples were not significantly different from the criterion and provided similar associations with body fat characteristics. Thus, a 1-min resting HRV recording preceded by a 1-min stabilization period seems to be a suitable alternative to criterion measures of 5 min preceded by a 5-min stabilization period. These shortened procedures may facilitate routine resting HRV tracking in tactical populations which may be useful for status-monitoring purposes.

## Figures and Tables

**Table 1:** Comparison values and statistics between criterion and 1-min segments.

Variable	Mean ± SD	*p*	Effect Size	Typical Error	ICC (95% CI)	Bias ± 1.96 SD
RR Criterion (ms)	966.4 ± 86.4	-	-	-	-	-
RR 0–1 (ms)	968.6 ± 86.7	0.99	0.03	28.3	0.95 (0.88, 0.97)	−2.3 ± 78.4
RR 1–2 (ms)	969.0 ± 86.9	0.99	0.03	21.0	0.97 (0.94, 0.99)	−2.7 ± 58.1
RR 2–3 (ms)	963.6 ± 87.1	0.99	−0.03	20.4	0.97 (0.94, 0.99)	2.7 ± 56.7
RR 3–4 (ms)	965.8 ± 90.0	0.99	−0.01	21.0	0.97 (0.94, 0.99)	0.5 ± 58.2
RR 4–5 (ms)	962.2 ± 86.4	0.98	−0.05	13.8	0.99 (0.97, 0.99)	4.2 ± 38.3
RMSSD Criterion (ms)	58.2 ± 21.5	-	-	-	-	-
RMSSD 0–1 (ms)	66.1 ± 24.5	0.14	0.34	15.3	0.85 (0.64, 0.93)	−7.9 ± 30.0
RMSSD 1–2 (ms)	63.7 ± 27.1	0.78	0.22	10.3	0.89 (0.77, 0.95)	−5.5 ± 28.6
RMSSD 2–3 (ms)	58.8 ± 25.0	0.99	0.03	8.4	0.93 (0.86, 0.97)	−0.6 ± 23.2
RMSSD 3–4 (ms)	58.5 ± 21.8	0.99	0.01	9.7	0.89 (0.77, 0.95)	−0.3 ± 26.8
RMSSD 4–5 (ms)	60.9 ± 24.9	0.89	0.12	6.8	0.95 (0.90, 0.98)	−2.7 ± 18.8
RMSSD: RR Criterion	6.0 ± 2.1	-	-	-	-	-
RMSSD: RR 0–1	6.8 ± 2.4	0.16	0.35	1.1	0.83 (0.60, 0.92)	−0.8 ± 3.1
RMSSD: RR 1–2	6.5 ± 2.6	0.99	0.21	1.0	0.88 (0.74, 0.94)	−0.5 ± 2.9
RMSSD: RR 2–3	6.0 ± 2.5	0.99	0.00	0.9	0.91 (0.82, 0.96)	−0.1 ± 2.5
RMSSD: RR 3–4	6.0 ± 2.2	0.99	0.00	1.1	0.86 (0.71, 0.94)	−0.0 ± 3.0
RMSSD: RR 4–5	6.3 ± 2.5	0.99	0.13	0.7	0.95 (0.89, 0.98)	−0.3 ± 1.9
SDNN Criterion (ms)	57.8 ± 18.9	-	-	-	-	-
SDNN 0–1 (ms)	59.3 ± 23.3	0.99	0.07	11.3	0.84 (0.65, 0.92)	−1.4 ± 31.4
SDNN 1–2 (ms)	57.8 ± 21.7	0.99	0.00	10.3	0.86 (0.69, 0.93)	0.0 ± 28.6
SDNN 2–3 (ms)	51.0 ± 21.3	0.13	−0.34	9.2	0.86 (0.66, 0.94)	6.8 ± 25.4
SDNN 3–4 (ms)	55.2 ± 20.0	0.99	−0.13	8.7	0.89 (0.76, 0.95)	2.6 ± 24.1
SDNN 4–5 (ms)	56.0 ± 21.6	0.99	−0.09	9.0	0.89 (0.77, 0.95)	1.9 ± 24.9
SDNN: RR Criterion	5.9 ± 1.8	-	-	-	-	-
SDNN: RR 0–1	6.1 ± 2.2	0.99	0.09	1.1	0.83 (0.63, 0.92)	−0.1 ± 3.0
SDNN: RR 1–2	5.9 ± 2.1	0.99	0.00	1.0	0.83 (0.64, 0.92)	0.0 ± 2.9
SDNN: RR 2–3	5.2 ± 2.1	0.13	−0.36	0.9	0.84 (0.60, 0.93)	0.7 ± 2.6
SDNN: RR 3–4	5.7 ± 1.9	0.99	−0.11	0.9	0.86 (0.71, 0.94)	0.3 ± 2.5
SDNN: RR 4–5	5.8 ± 2.1	0.99	−0.05	0.9	0.86 (0.71, 0.94)	0.1 ± 2.5

ICC = intra-class correlation; SD = standard deviation; RMSSD = root-mean square of successive RR interval differences; SDNN = standard deviation of normal-to-normal RR intervals.

**Table 2:** Correlation coefficients between heart rate variability parameters and markers of fitness and body composition.

Variable	Body Fat (%)	Fat Mass (kg)	Fat-Free Mass (kg)
RR Criterion (ms)	−0.321	−0.453 *	−0.112
RR 0–1 (ms)	−0.407 *	−0.506 **	−0.116
RR 1–2 (ms)	−0.458 *	−0.555 **	−0.147
RR 2–3 (ms)	−0.405 *	−0.500 **	−0.087
RR 3–4 (ms)	−0.371	−0.471 *	−0.066
RR 4–5 (ms)	−0.371	−0.492 **	−0.128
RMSSD Criterion (ms)	−0.420 *	−0.487 *	−0.149
RMSSD 0–1 (ms)	−0.401 *	−0.364	−0.052
RMSSD 1–2 (ms)	−0.489 **	−0.445 *	−0.042
RMSSD 2–3 (ms)	−0.377	−0.384 *	−0.103
RMSSD 3–4 (ms)	−0.351	−0.320	−0.051
RMSSD 4–5 (ms)	−0.310	−0.395 *	−0.217
RMSSD: RR Criterion	−0.389 *	−0.427 *	−0.126
RMSSD: RR 0–1	−0.319	−0.250	−0.009
RMSSD: RR 1–2	−0.435 *	−0.383 *	−0.008
RMSSD: RR 2–3	−0.326	−0.307	−0.070
RMSSD: RR 3–4	−0.272	−0.210	−0.015
RMSSD: RR 4–5	−0.256	−0.314	−0.182
SDNN Criterion (ms)	−0.442 *	−0.515 **	−0.114
SDNN 0–1 (ms)	−0.426 *	−0.416 *	−0.062
SDNN 1–2 (ms)	−0.523 **	−0.479 *	−0.020
SDNN 2–3 (ms)	−0.417 *	−0.401 *	−0.034
SDNN 3–4 (ms)	−0.445 *	−0.472 *	−0.072
SDNN 4–5 (ms)	−0.252	−0.385 *	−0.207
SDNN: RR Criterion	−0.418 *	−0.460 *	−0.086
SDNN: RR 0–1	−0.367	−0.337	−0.041
SDNN: RR 1–2	−0.454 *	−0.386 *	−0.018
SDNN: RR 2–3	−0.374	−0.333	−0.001
SDNN: RR 3–4	−0.391 *	−0.398 *	−0.053
SDNN: RR 4–5	−0.203	−0.313	−0.180

RMSSD = root-mean square of successive RR interval differences; SDNN = standard deviation of normal-to-normal RR intervals. * = *p* < 0.05; ** = *p* < 0.01.
